# Derivation and validation of the Pediatric Community‐Acquired Pneumonia Severity (PedCAPS) score: A prospective cohort study

**DOI:** 10.1002/jhm.70220

**Published:** 2025-11-21

**Authors:** Todd A. Florin, Ron Reeder, Lilliam Ambroggio, Richard M. Ruddy, Samir S. Shah, Allison Cator, Matthew J. Lipshaw, Geoffrey A. Capraro, Laura F. Sartori, Amy Y. Cheng, Leah Tzimenatos, Patrick S. Walsh, Claudia R. Morris, Chris A. Rees, Son H. McLaren, Tamar R. Lubell, Chari D. Larsen, Justin Moher, Eileen J. Klein, Shubhada Hooli, Nathan Kuppermann, Pediatric Emergency Care Applied Research Network (PECARN) PedCAPS Investigators

**Affiliations:** ^1^ Department of Pediatrics, Division of Pediatric Emergency Medicine Northwestern University Feinberg School of Medicine, Ann and Robert H. Lurie Children′s Hospital of Chicago Chicago Illinois USA; ^2^ Department of Pediatrics University of Utah School of Medicine Salt Lake City Utah USA; ^3^ Sections of Emergency Medicine and Hospital Medicine, Children′s Hospital Colorado, Department of Pediatrics University of Colorado School of Medicine Aurora Colorado USA; ^4^ Division of Emergency Medicine, Cincinnati Children′s Hospital Medical Center, Department of Pediatrics University of Cincinnati College of Medicine Cincinnati Ohio USA; ^5^ Divisions of Hospital Medicine and Infectious Diseases, Cincinnati Children′s Hospital Medical Center, Department of Pediatrics University of Cincinnati College of Medicine Cincinnati Ohio USA; ^6^ Departments of Emergency Medicine and Pediatrics University of Michigan Medical School and Children′s Emergency Services, C.S. Mott Children′s Hospital Ann Arbor Michigan USA; ^7^ Departments of Emergency Medicine and Pediatrics Hasbro Children′s Hospital/Warren Alpert Medical School of Brown University Providence Rhode Island USA; ^8^ Division of Emergency Medicine, Children′s Hospital of Philadelphia, Department of Pediatrics, Perelman School of Medicine University of Pennsylvania Philadelphia Pennsylvania USA; ^9^ Division of Pediatric Emergency Medicine, Department of Pediatrics University of Texas Southwestern Medical Center Dallas Texas USA; ^10^ Department of Emergency Medicine University of California, Davis School of Medicine Sacramento California USA; ^11^ Department of Pediatrics, Section of Pediatric Emergency Medicine Medical College of Wisconsin Milwaukee Wisconsin USA; ^12^ Division of Pediatric Emergency Medicine Emory University School of Medicine Atlanta Georgia USA; ^13^ Department of Emergency Medicine Children′s Healthcare of Atlanta Atlanta Georgia USA; ^14^ Division of Pediatric Emergency Medicine, Department of Emergency Medicine Columbia University Vagelos College of Physicians and Surgeons New York New York USA; ^15^ Division of Pediatric Emergency Medicine, Department of Pediatrics University of Utah School of Medicine Salt Lake City Utah USA; ^16^ Division of Emergency Medicine, Department of Pediatrics University of Washington School of Medicine Seattle Washington USA; ^17^ Department of Pediatrics, Division of Pediatric Emergency Medicine Texas Children′s Hospital, Baylor College of Medicine Houston Texas USA; ^18^ Departments of Pediatrics and Emergency Medicine GW School of Medicine and Health Sciences and Children′s National Hospital Washington District of Columbia USA

## Abstract

**Introduction:**

Community‐acquired pneumonia (CAP) is a frequent and costly cause of pediatric emergency department (ED) visits and hospitalizations. Previous prognostic tools for CAP are limited by small samples, single‐center or retrospective designs, lack of generalizability to ED settings, lack of biomarkers, or limited objective data. To overcome these limitations, we will derive and externally validate a prediction rule for pediatric CAP severity in a large, multicenter prospective cohort.

**Methods:**

This is a prospective cohort study of children 3 months to 18 years old with CAP who present to EDs within the Pediatric Emergency Care Applied Research Network. Enrollment began 8/2023 and will end 7/2027. We exclude children with recent hospitalizations and chronic conditions (e.g., immunosuppression). A follow‐up survey and record review is completed 8–15 days after the visit. Blood and nasal specimens are obtained to evaluate the role of C‐reactive protein, procalcitonin, proadrenomedullin, and viral detection in severity prediction. The primary outcome is severity (three‐tiered outcome of mild, moderate, or severe CAP) within 7 days of ED presentation. Model derivation will occur in ~4000 children from 7 EDs over 2 years. External validation will occur in a distinct cohort of at least 2000 children from 7 different EDs. Penalized regression, recursive partitioning, and machine learning will be used in model development.

**Discussion:**

At study completion, we will have a validated CAP severity prediction rule well‐positioned for implementation and further evaluation. We will also understand the role of specific biomarkers in predicting outcomes in children with CAP.

## INTRODUCTION

Community‐acquired pneumonia (CAP) leads to two million pediatric healthcare visits annually in the United States (US) and accounts for 1%–4% of all emergency department (ED) visits in children. Additionally, CAP is among the most common reasons for pediatric hospitalizations from the ED in the US and contributes the most total days of antibiotic use in US children′s hospitals.^1^, ^2^, ^3^, ^4^, ^5^, ^6^

The decision to manage children as outpatients or inpatients is one of the most important decisions in the management of CAP.^7^ Hospitalization poses substantial, and potentially unnecessary, burdens on children, families, and the healthcare system. In previously healthy children, outpatient treatment is preferable for children at low risk of severe illness. Yet, hospitalization rates vary widely by region and hospital, even after adjustment for illness severity, suggesting that admission criteria are not consistent across providers or institutions.^8^, ^9^ In a prospective cohort study of 1142 children presenting to the ED with suspected CAP, 40% of those hospitalized were discharged within 24 h. Of those, 72% did not require oxygen, 85% did not require intravenous (IV) fluids, and none required invasive respiratory support.^10^ Hospitalization is likely unnecessary for many children, exposing them to potential nosocomial infections, medical errors, time lost from school/work, anxiety, and cost. On the other hand, severe complications of CAP, although uncommon,^10^ can be devastating and may be preventable with earlier identification and targeted interventions.

Accurate assessment and prognostication of disease severity is critical to providing appropriate care to children with CAP. Management decisions, however, are currently based on nonspecific physical examination findings,^11^, ^12^, ^13^ radiographic images,^14^, ^15^, ^16^ and conventional laboratory markers that do not accurately assess disease severity.^17^, ^18^ The Pediatric Infectious Diseases Society (PIDS)/Infectious Diseases Society of America (IDSA) pediatric CAP guideline extrapolated illness severity criteria from adult guidelines.^19^ These criteria were not derived in children and do not account for the distinct features of pediatric CAP, including differences in etiology and severity. When applied to children, these criteria demonstrate only fair ability to predict disease severity.^20^ Another study found that ED clinicians had only fair ability to discriminate between children developing severe CAP from those who did not. The sensitivity of clinician impression alone to predict severe CAP ranged from 14% to 57%.^21^ In addition, of those children who developed severe CAP, more than 60% were initially classified as having a <10% predicted risk of these outcomes by ED clinicians. The limitations of clinician gestalt, the extrapolating of adult criteria to children, and the drawbacks of unnecessary hospitalization or delayed therapies, suggest a critical need for evidence‐based prognostic tools to complement clinical judgment in the management of children with CAP. The PIDS/IDSA pediatric CAP guideline emphasizes this critical gap as key to future research.^19^

While the use of risk prediction rules in adults with CAP decreases mortality and hospitalization rates, and are used to guide antibiotic decision‐making,^22^, ^23^ there are no widely validated pediatric CAP severity scores for use in the ED. Three pediatric prediction rules have been published in well‐resourced health systems. The first was derived in 2319 hospitalized children with CAP and consisted of 9‐10 prospectively obtained variables with bootstrapped c‐indices of 0.76–0.77, indicating acceptable discrimination.^17^ As these patients were already hospitalized, these rules cannot be extrapolated to the ED, where disposition decisions are made. In addition, definitions of mild and moderate CAP for that rule were largely based on care locations (e.g., general ward, intensive care unit) rather than objective outcomes. The second rule was derived and internally validated in 1128 children who presented to a single pediatric ED with suspected CAP.^10^ The rule consisted of seven variables and had a bootstrapped c‐index of 0.81 and outstanding calibration. Limitations of this rule were its inclusion of clinically diagnosed CAP regardless of the presence of radiographic pneumonia, relatively few patients with severe outcomes, and questions about generalizability given its single‐center derivation. The third analysis included rules derived in a cohort of more than 2000 children 3 months to 14 years old across 73 EDs in 14 countries.^24^ This rule included 8 variables and had a bootstrapped c‐index of 0.82. Limitations include lack of validation, lack of biomarker data, and its use of a convenience sample.

None of these existing pediatric CAP prediction models fully examined the role of biomarkers in risk prediction. Studies demonstrate that biomarkers, such as C‐reactive protein (CRP), procalcitonin (PCT), and midregional proadrenomedullin (proADM), may be associated with disease severity in children and adults with CAP, and enhance the predictive ability of CAP risk prediction models in adults with CAP.^25^, ^26^, ^27^, ^28^ Although these associations have been explored, the impact of adding these biomarkers to pediatric CAP severity risk prediction models remains unclear.

Accurate predictions of clinical outcomes and disease severity would enhance evidence‐based disposition decisions in the ED, and allow for timely initiation of intensive therapies for those at highest risk, while minimizing resource use for those at low risk. We will overcome the limitations of prior prediction rules by deriving and externally validating a prediction rule for CAP severity in children in a large multicenter cohort of pediatric EDs. Our specific aims are to: (1) derive a severity risk prediction rule in a multicenter cohort of children presenting to the ED with CAP; (2) externally validate our severity risk prediction rule in children with CAP; and (3) evaluate the ability of selected biomarkers (CRP, PCT, proADM, viral testing) to improve predictive accuracy of a purely clinical risk prediction rule. At the end of this study, we expect to have a validated risk prediction rule well‐positioned for implementation into clinical care at the bedside and for use in future pediatric CAP research.

## METHODS AND ANALYSIS

### Study overview

This study is a prospective cohort study using a convenience sample of children 3 months up to their 18th birthdays who present to one of 14 EDs that participate in the Pediatric Emergency Care Applied Research Network (PECARN) in the United States.

### Study population

#### Inclusion criteria

Children 3 months up to 18 years old with CAP are included. We exclude children younger than 3 months, as young infants with fever and lower respiratory diseases have distinct diagnostic and therapeutic approaches. Likewise, we exclude those 18 years and older, as these patients are included in established adult CAP rules. We include children with: (1) signs and symptoms consistent with lower respiratory tract infection (defined as at least one of the following: new or different cough or sputum production, chest pain, dyspnea, tachypnea, or abnormal auscultatory findings (e.g., crackles),^29^ (2) fever (defined as >38.0 C) in the ED or measured or tactile fever at home in the prior 48 h, (3) an ED clinician diagnosis of CAP, and (4) suspicion of pneumonia based on chest radiography (CXR), if performed, as interpreted by the attending ED physician or radiologist.

#### Exclusion criteria

The goal of this study is to predict CAP severity in children without substantial comorbidities that would predispose them to very severe pneumonia or to pathogens not typically encountered in children without those comorbidities. Therefore, we exclude children who were hospitalized in the 30 days before the study ED visit to avoid the potential for including children with hospital‐acquired pneumonias, or with chronic pulmonary diseases (e.g., cystic fibrosis, chronic lung disease of prematurity, tracheostomy‐dependence), physiologically‐significant cardiac disease (not including functionally insignificant murmurs), immunosuppression or immunodeficiency (e.g., chronic corticosteroid use, oncologic process on chemotherapy), sickle cell disease, or neuromuscular disorders affecting respiration. Children with asthma are included. We also exclude those with aspiration pneumonia, enrollment within 30 days before the ED visit (to ensure distinct episodes of CAP), and those transferred from other EDs or hospitals (as predictors from those visits would not be captured).

### Study procedures

#### Participant screening and consent

Clinicians or research staff enroll children 24 h per day, 7 days per week to ensure consecutive enrollment to minimize the potential for selection bias. Enrollment typically is led by research staff when they are present in the ED and by clinicians when research staff are not present (e.g., overnight). An information sheet describing the study is provided to the parents/guardians of enrolled children. The trained research staff provide additional support for enrollment and study procedures, particularly around specimen collection. We have obtained a waiver of informed consent for screening, enrollment, and follow‐up procedures by the study single institutional review board (IRB) at the University of Utah. The waiver of written consent meets 45CFR46.116 requirements, due to the minimal risk of this non‐interventional study and to avoid potential enrollment bias that would risk the scientific integrity of the study.

#### Biospecimen collection

Participants enrolled during hours when research staff are available are approached to obtain nasal or nasopharyngeal (NP) swabs and blood for biomarker measurements. Informed consent is obtained for specimen collection and biomarker assays. Given the fast timing of specimen collection for clinical care in the ED and our desire to minimize burden on participants and clinical staff, we can obtain initial verbal consent from parents/guardians (and assent from participants able to provide it) to obtain specimens. At an appropriate time, research staff discusses the study further with the participants and their parents/guardians and written informed consent is documented.

Blood draws occur in three possible ways: (1) blood can be drawn concurrently with that drawn for clinical care; (2) via a separate phlebotomy blood draw for research; or (3) via existing intravenous catheters. Up to 5 mL of blood is frozen and stored for CRP, PCT, and proADM assays, in addition to future analyses. The NP swab will be used for comprehensive viral and atypical bacterial multiplex polymerase chain reaction (PCR) analyses to account for the role of microbiological etiology in modeling. NP swabs are collected using a flocked swab, placed in universal transport medium, and frozen at −80°C. All frozen specimens are shipped to and stored at Lurie Children′s Hospital. All specimens not used for this study will be stored in a biorepository for potential future analyses.

#### Data collection

As this is an observational study, all clinical care is at the discretion of the treating physicians. ED clinicians complete standardized data collection forms to record eligibility, patient history, and physical examination findings, and their rationale for disposition decisions. Research staff also abstract medical record data from the electronic health record, including patient history, vital signs, laboratory and imaging results, and information pertaining to all treatments and/or medications given during the enrollment visit and associated hospital admissions. To measure clinician gestalt, ED clinicians are asked to select a number along a scale from 0% to 100% for their predicted probability that the child will develop moderate or severe CAP at a time when all ED‐based data are available for clinical decision‐making.^21^ For a subset of participants, a second clinician performs a physical examination within 60 min of the initial examination to assess the inter‐rater reliability of predictors considered for the clinical prediction models.

#### Participant follow‐up

A standardized follow‐up survey of patients discharged from the ED is performed 8–15 days after the index ED visit to ascertain outcomes that may be missed by medical record review. This survey is completed via a text message link to a mobile‐friendly RedCAP form. Follow‐up data includes return to medical care, changes in antibiotics, and progression of CAP. The research staff review the medical record between 8 and 30 days after the ED visit to determine if discharged patients returned for care within 7 days of the study visit.

### Predictor variables

Candidate predictors of CAP severity (Table 1) were selected through an extensive literature review, including a published systematic review, consensus from a Delphi panel of experts, and from prior studies evaluating features associated with clinical outcomes in children with CAP.^30^, ^31^ Definitions for all predictor variables are outlined in the study manual of operations that was distributed to all sites before the commencement of enrollment. All predictors are assessed before the occurrence of the outcome. Thus, if a patient presents to the ED with severe CAP already present, they will not be included in prediction modeling.

**Table 1:** Candidate predictors of disease severity in pediatric community‐acquired pneumonia.

*Demographics*
Age
*Historical factors*
Symptom duration
Maximum temperature at home
Shortness of breath/difficulty breathing
No oral intake in >12 h
Vomiting/feeding refusal
Antibiotic exposure
Prior pneumonia hospitalization
Comorbidities (e.g., asthma)
*Physical examination*
Vital signs (e.g., heart rate, respiratory rate, systolic blood pressure, temperature)
Oxygen saturation
Clinical appearance
Mental status
Capillary refill
Chest auscultatory findings (e.g., wheezes, rales, rhonchi)
Work of breathing (e.g., chest indrawing, grunting, nasal flaring)
*Imaging*
Chest radiograph findings
Chest ultrasound findings
Chest computed tomography findings

### Outcome measures

The primary outcome will be CAP severity, represented by a three‐tiered composite outcome, with outcomes defined as occurring within 7 days of the index ED visit (Table 2). *Severe CAP* will be defined as the development of empyema or effusion requiring drainage procedures, intensive care unit admission 24 h or more in duration, respiratory failure requiring positive pressure ventilation (invasive or noninvasive), severe sepsis/septic shock, receipt of vasoactive infusions, receipt of extracorporeal membrane oxygenation (ECMO), or death. *Moderate CAP* will be defined as not meeting criteria for severe CAP plus any of the following occurring within 7 days of the index ED visit: receipt of supplemental oxygen on an inpatient unit, documented valid and sustained room air oxygen saturation of <91%, hospital admission with length of stay (LOS) greater than or equal to 36 h, or any hospitalization that occurs during a return to the ED after initial discharge home after index visit. Hospital length of stay of 36 h was selected as it was the median LOS in a prior prediction model for pediatric CAP severity^10^ and was thought to the be the cut point after which our investigators felt a hospitalization would be warranted. *Mild CAP* will be defined as CAP treated in the outpatient setting or not meeting criteria for moderate or severe CAP. Because predictive models aim to identify patients for whom an outcome is likely to occur, rather than to identify outcomes which are already present, we will exclude from modeling patients who meet the severe CAP definition in the ED (before the disposition decision is made).

**Table 2:** Primary outcome: Disease severity.

Mild
Community‐acquired pneumonia (CAP) not meeting conditions for moderate or severe CAP
*Moderate*
Conditions for severe CAP are not met AND any of the following occur within 7 calendar days after index ED presentation:
Supplemental oxygen on an inpatient unit; or
Documented valid room air oxygen saturation of <91%; or
Hospital admission with length of stay ≥36 h; or
Any hospital admission as a result of a return to the ED after initial discharge home
*Severe*
CAP with:
Empyema or effusion requiring drainage procedures; or
Intensive care unit admission >24 h in duration; or
Diagnosis of severe sepsis/septic shock; or
Respiratory failure requiring positive pressure ventilation (invasive or noninvasive); or
Receipt of vasoactive infusions; or
Receipt of extracorporeal membrane oxygenation (ECMO); or
Death

A pragmatic secondary outcome is the actual hospitalization decision by the treating clinicians. This outcome will allow us to compare the risk prediction rule for CAP severity with the actual decision for hospitalization. An additional secondary outcome will be length of stay for hospitalized patients.

### Data analysis

#### Sample size and power

For rule derivation, we estimated the necessary sample size using two approaches. First, we evaluated the area under the receiver operating characteristic curve (AUROC), compared with clinician gestalt (Table 3). In the single‐center study serving as the foundation for this study, 62.8% of children developed mild CAP, 32.9% moderate, and 4.3% severe.^10^ Prior work suggests that the AUROC of clinician gestalt for predicting severe CAP in children ranges from 0.69 to 0.75.^21^ Our primary comparison is mild versus moderate/severe. Assuming an AUROC of clinician gestalt is ∼0.7 (i.e., null hypothesis) and the prevalence of moderate or severe CAP is 37.2%, 317 children would be needed to demonstrate an improvement in the model AUROC from 0.7 to 0.8 (the necessary AUROC threshold for a prediction rule to be considered to have excellent discriminatory ability over gestalt^32^) and 1220 would be needed to show a difference from 0.75 to 0.8. For a secondary evaluation of discriminating severe from non‐severe CAP, assuming a prevalence of severe CAP of 4.3%, 1977 children would need to be enrolled to detect an AUROC of at least 0.8 for the model to distinguish severe from non‐severe CAP.

**Table 3:** Sample size estimates.

	Moderate or severe disease				Severe disease			
Prediction rule AUROC	*Clinical Gestalt AUROC*				*Clinical Gestalt AUROC*			
	0.6	0.65	0.7	0.75	0.6	0.65	0.7	0.75
0.75	153	339	1320	—	907	2047	8140	—
0.8	83	145	317	1220	512	884	1977	7767
0.85	51	78	132	285	302	488	837	1837
0.9	32	46	67	113	209	279	442	744

*Note*: Assumptions include two‐sided 5% type 1 error, 90% power, 32.9% moderate CAP prevalence, 4.3% severe CAP prevalence.^10^

Abbreviation: AUROC, area under the receiver operating characteristic curve.

Second, simulation studies suggest that a minimum of 10 events per variable under consideration is a conservative estimate in model development to avoid overfitting and generate reliable estimates.^33^, ^34^ As our outcome is ordinal, we powered conservatively on the least frequent outcome (i.e., severe CAP). Assuming 4% will develop severe CAP, we will need to include 2000–3500 children in the rule derivation to capture at least 80–90 who will develop severe CAP. For moderate or severe CAP, with an assumed prevalence of 37.2%, we would require ∼1000 participants. These estimates would allow for a rule with 8–10 potential predictors. This is consistent with prior CAP prediction rules^10^, ^17^, ^24^ and with practical considerations to avoid an overly complicated rule.

Biomarkers will be collected throughout all 4 years of both the derivation and validation phases. We will compare the AUROC of the clinical prediction model with and without biomarkers. Assuming an AUROC of 0.80 for the model without biomarkers, an 80% correlation between models, and a 37.2% event rate for moderate or severe outcomes, a sample of 800 participants with biomarker data will provide 80% power to detect a difference in AUROC of 0.033 (e.g., from 0.800 to 0.833).

#### Statistical modeling and analysis

Models will be developed using seven key steps in prediction rule development: data inspection, coding of predictors, model specification, model estimation, measuring model performance, evaluating model validation, and model presentation.^35^ All prediction modeling will be performed and reported following the Transparent Reporting of a Multivariable Prediction Model for Individual Prognosis or Diagnosis (TRIPOD) guideline.^36^ Multiple approaches will be used to develop potential models. We will use penalized ordinal logistic regression models to examine the independent association of each potential predictor with the outcome. A final model based on the independent association will be determined using step‐down backward variable selection with bootstrap validation, lasso regression, ridge regression, or elastic net regression. We will compare various model performance using criteria such as c‐index, calibration, *R* ^2^ and Akaike information criteria (AIC) to select the best performing model. In addition to a regression‐based approach, we will explore Classification and Regression Trees (CART) and machine learning (ML) approaches.

We will evaluate four key measures to assess the external validity of the prediction rule.^35^ The first two are related to calibration, assessed graphically with the predictions on the horizontal axis and observed values on the vertical axis. The first measure is *calibration‐in‐the‐large*, the intercept of the calibration slope, which comprises the mean of all predicted risks with the mean observed risk. This measure evaluates whether predictions are systematically too low or high. The second measure, the *calibration slope*, evaluates if predictions are too extreme. A slope close to 1 indicates a well calibrated model. The third measure is *discrimination*, as measured by the c‐index (or AUROC curve). The fourth measure, *decision curve analysis*, assesses the clinical usefulness of the validated rule.^37^ A decision threshold represents the probability at which the expected benefit of intervention equals the expected harm. Because this threshold varies by patient and clinician preferences, decision curve analysis will assess a range of thresholds. For each threshold, sensitivity, specificity, and net benefit will be calculated to quantify the model′s clinical value.

After the clinical prediction rule is derived and validated, we will examine the improvement in the clinical rule′s performance using biomarkers. First, the predicted probability of moderate or severe CAP will be calculated for each participant using the validated risk prediction model. The log‐odds will then be included as a covariate in a logistic regression model that also includes CRP, PCT, proADM, and/or pathogen results from respiratory PCR assays on the NP swabs and bacterial cultures, when obtained. Performance of the biomarker‐enhanced model will be compared with the original clinical model calibrated to the same cohort. The net reclassification improvement (NRI) will be calculated to quantify the extent of reclassification of patients into different risk groups based on biomarker results.^38^ Alternative measures, such as the weighted NRI or likelihood ratio tests, will be explored.^39^

#### Ethics and dissemination

This study poses minimal risk to participating children and their families. Ethics approval has been obtained at all participating sites. The University of Utah IRB is serving as the central IRB. Patients receive standard care in the ED. Participation in the study does not impact or restrict care in the ED or hospital. A small potential risk exists around disclosure of confidential information. A waiver of informed consent has been obtained for clinical data collection. All parents/guardians will provide written or verbal informed consent/assent for biospecimen collection and will have the ability to withdraw at any time without explanation. Results will be disseminated at national and international conferences and through peer‐reviewed research publications.

#### Patient and public involvement

This study was planned without patient involvement. Patients were not invited to comment on the study design and were not consulted to develop patient‐relevant outcomes or interpret the results. Patients were not invited to contribute to the writing or editing of this document for readability or accuracy. We anticipate that parents and patients will play an essential role in the implementation of the clinical prediction models that are developed as part of this study.

## DISCUSSION

This study will produce an externally validated risk prediction model for pediatric CAP severity that will be well‐positioned for implementation and further evaluation. Our study has several key strengths. First, it will be one of the largest prospective cohorts of children with CAP to evaluate clinical outcomes and disease severity. With more than 4000 children in the derivation cohort and at least 2000 in the validation cohort, we will have sufficient power and heterogeneity to ensure a precise, generalizable, and valid clinical prediction model. Second, both the derivation and external validation cohorts are enrolled in the ED, where most hospitalization decisions are made. This will allow for rapid implementation of the model in the most relevant setting for its use. In addition, by enrolling a validation cohort in distinct sites, in a distinct time period, with distinct patients from the derivation cohort, this model will be ready for implementation at study completion. Third, our composite outcome measure was developed to represent reasons a patient may potentially require hospitalization or intensive care, rather than relying entirely on a clinician′s decision to hospitalize. Furthermore, unlike prior work, we did not combine outpatients and inpatients into a single stratum. Finally, we will be able to incorporate biomarkers into the prediction model to improve its accuracy and precision.

Our study also has several anticipated limitations. First, this study is being conducted in the United States at pediatric referral centers. Thus, it may not be fully generalizable to community centers or to settings that are less resourced to care for children. This will be an important aspect of studying the implementation of these models. Second, although we enroll 24 h per day, 7 days per week, it is possible that we will miss more patients during overnight hours when research staff are not available to assist clinicians with enrollment. Third, due to challenges of collecting biospecimens for research during overnight hours, we will not have biospecimen data on all enrolled patients.

Objective pediatric CAP prognostic models will improve risk assessment, rapid treatment, and appropriate resource allocation, including hospitalization and disposition decisions. This innovative departure from the status quo will shift care based on clinician gestalt to a targeted, precision‐oriented approach in which risk can be quantified for each patient. Implementation of this prediction rule will improve clinical outcomes, make care more efficient and precise, and guide future research by targeting those at risk for severe CAP to allow for focused, early interventions to prevent disease progression, while reducing unnecessary hospitalizations and resource use in those at low risk.

## CONFLICT OF INTEREST STATEMENT

The authors declare no conflicts of interest.
